# Real‐world, long‐term treatment patterns of commonly used biologics in Canadian patients with moderate‐to‐severe chronic plaque psoriasis

**DOI:** 10.1111/1346-8138.16214

**Published:** 2021-11-07

**Authors:** Melinda J. Gooderham, Charles Lynde, Irina Turchin, Miriam Avadisian, Melanie Labelle, Kim A. Papp

**Affiliations:** ^1^ Probity Medical Research Inc. Waterloo Ontario Canada; ^2^ SkiN Centre for Dermatology Peterborough Ontario Canada; ^3^ Queen’s University Kingston Ontario Canada; ^4^ Lynderm Research Inc. Markham Ontario Canada; ^5^ University of Toronto Toronto Ontario Canada; ^6^ Brunswick Dermatology Center Fredericton New Brunswick Canada; ^7^ Department of Medicine Dalhousie University Halifax Nova Scotia Canada; ^8^ AbbVie Corporation Saint‐Laurent QC Canada; ^9^ K. Papp Clinical Research Waterloo Ontario Canada

**Keywords:** biologics, drug survival, moderate‐to‐severe plaque psoriasis, real‐world data, treatment pattern

## Abstract

Real‐world and long‐term data on biologic treatment changes – including switching, discontinuation, dose escalation, and interval change (both increasing and decreasing) – are required to understand treatment patterns for psoriasis (PsO) in Canada. The study objectives were to evaluate the time to first biologic treatment change and to document these changes in Canadian patients with moderate‐to‐severe chronic plaque PsO. Charts from 13 Canadian sites were queried retrospectively (2005–2019); a period covering all biologic classes commonly used for PsO in Canada. Included were patients diagnosed with, and currently using biologics for, moderate‐to‐severe chronic plaque PsO. Time to first treatment change, nature of treatment change, number of lines of treatment, proportion of patients on each drug, and drug survival were collected. Based on 1149 medical charts, adalimumab had the longest time to first treatment change (49.3 months; 95% confidence interval, 37.4–67.4). Approximately half of the patients had a treatment change, and nearly 75% of these changes were either an interval change or a biologic switch. Lack of efficacy was the most prevalent primary reason for biologic switch (67.3%), whereas 6.7% of patients switched due to adverse events. Drug survival for etanercept and infliximab was approximately twice as long for patients who had dose optimization (i.e., dose escalation or interval change) than patients without dose optimization. The survival curve of adalimumab was similar to the one of ustekinumab after 48 months of treatment, showing approximately 60% of patients remaining on treatment after 132 months, with or without dose optimization. Assessing treatment patterns of all commonly used biologics for moderate‐to‐severe chronic plaque PsO in Canada between 2005 and 2019 showed that approximately half of the patients required a treatment change (mainly interval change or biologic switch) while the other half remained on treatment.

## 1 INTRODUCTION

Biologics have advanced our understanding and the treatment of psoriasis (PsO) therapy due to their efficacy and precise mechanisms of action.^1^, ^2^, ^3^ Despite the demonstrated efficacy of biologics, ≥30% of patients show an inadequate response to these agents.^1^, ^4^, ^5^ Treatment modifications, including dose escalations, dose reductions, switches, discontinuations, and restarts, are to be expected in the management of PsO.^6^, ^7^, ^8^

In clinical practice, modifications of dosing regimens,^9^, ^10^, ^11^, ^12^, ^13^ intermittent therapy, or interruption followed by retreatment^14^, ^15^ have been reported to impact treatment effectiveness. Biologic switches in the treatment of PsO have been evidenced to be mostly due to a lack of efficacy, to adverse events (AE) to a lesser extent, or to efforts to achieve better clinical response.^6^, ^10^, ^15^, ^16^, ^17^, ^18^, ^19^ Specific to Canadian real‐world practices, off‐label regimens are less likely to include biologics dose reductions or interval increases compared with other practices worldwide such as European practices.^11^

Drug survival, defined as the duration of time from therapy initiation to discontinuation, is a proxy measure for drug effectiveness, safety, and tolerability. Predictors of biologic drug survival have been reported in specific studies as female sex,^20^ psoriatic arthritis (PsA),^21^, ^22^, ^23^ dose escalation,^24^, ^25^ and previous exposure to biologics.^21^ Gradual loss of efficacy has been shown to limit biologic drug survival,^26^ and several studies have reported ustekinumab as having the highest survival rate.^19^, ^25^, ^27^, ^28^, ^29^, ^30^, ^31^

Given the paucity of data on biologic treatment patterns for Canadian PsO patients, real‐world, long‐term data are needed, including data on recently approved biologics, although less extensive results are available on these biologics. The primary objective of this study was to evaluate retrospectively, in a real‐world setting, the time to first treatment change – defined as switching, discontinuation, dose escalation, and interval change (both increasing and decreasing) – for commonly used biologics in Canadian patients with moderate‐to‐severe chronic plaque PsO. As a secondary objective, these treatment changes were documented in terms of number, types and reasons for changes, sequence of agents used as well as drug survival. These results may help identify effective therapies to clear the skin of PsO patients while minimizing treatment changes.

## 2 METHODS

### 2.1 Study design and setting

This study was a Canadian, non‐interventional, retrospective chart review of moderate‐to‐severe chronic plaque PsO patients using biologics. Biologics included were from four classes: tumor necrosis factor (TNF)‐α inhibitors (etanercept, adalimumab, and infliximab), interleukin (IL)‐12/23 inhibitor (ustekinumab), IL‐17 inhibitors (secukinumab, ixekizumab, and brodalumab [receptor blocker]), and IL‐23 p19 inhibitor (guselkumab).

Retrospective data from 2005 to 2019 were obtained from 13 Canadian sites. The date range was selected to include the above‐mentioned biologics since their approval in Canada for treating PsO. Sites were selected based on chart availability and relevance of geographical representation. Intrinsic to the nature of this study was the variability in the number of years since approval for these biologics, thus leading to more patients having “ongoing treatments” with the most recent biologics approved, namely no first treatment change at the time of conducting the study. The study was conducted from July 2019 to June 2020.

### 2.2 Participants

Included in this study were charts of patients who were ≥18 years of age (or as per local legal age) at initiation of their first PsO biologic therapy and who were diagnosed with – and currently using biologics as primary indication for – moderate‐to‐severe chronic plaque PsO. In addition, the first therapy had to be initiated as per label at the starting dose, with the most recent therapy lasting ≥1 year. There were no exclusion criteria. The study was conducted in compliance with local laws and regulations and Good Pharmacoepidemiology Practices, and was approved by central and local ethic boards prior to initiation.

### 2.3 Variables and data sources

To minimize chart selection bias, sites were requested to pull charts from the latest consecutive patients seen and who fit the inclusion criteria. Of charts meeting the inclusion criteria, patient demographics, PsO history, and comorbidities were retrospectively collected as well as data on PsO treatments available since 2005: treatment name, dose prescribed, administration mode, dosing start and stop dates, and reason for discontinuation or treatment change, if any.

Time to treatment change was calculated based on the start and stop/change dates. Stop dates for ongoing treatments at the time of chart review were censored at the chart review date. If patients participated in clinical trial(s) for PsO during the period covered by this study, such period(s) was/were excluded from the study.

In this study, treatment changes were defined as either switching, discontinuation, dose escalation, or interval change. Interval changes included both increasing and decreasing, which were more likely to be interval decreases than the opposite in Canadian real‐world practices. Information on biologics on‐label dosing and year of market entry for PsO treatment in Canada is provided in Table S1.

### 2.4 Study size and statistical methods

Sample size considerations were based on time to first treatment change – the study primary end‐point. Using published drug survival times for biologic treatments in PsO (23–38 months),^20^, ^32^ time to first treatment change was assumed to be 30 months. Assuming a censor rate (proportion of patients for whom treatment change was not observed) of 30%, the sample size was calculated to provide an estimate of the median time to first treatment change within a pre‐defined confidence interval (CI) (≥23 and ≤39 months).

The primary end‐point was analyzed and plotted using Kaplan–Meier (KM) estimates (reported with 95% CI). The secondary end‐points, time to treatment change as second line of treatment, and drug survival – the number of months until drug discontinuation – were analyzed using the same KM estimates as for the primary end‐point. The number of biologics per patient was summarized as mean, median, standard deviation (SD), minimum, and maximum.

These analyses were performed on the full analysis set (FAS; n = 1149) population, defined as patients who met all inclusion criteria and had been treated for PsO with any of the biologics for at least 1 year. Patients whose most recent biologic treatment was <1 year, but the combined length of treatment for all biologics was ≥1 year were also included, even if not meeting protocol entry criterion. As a sensitivity analysis, the primary end‐point was analyzed using the per‐protocol set (PPS; n = 1059), defined as all patients of the FAS who met all inclusion criteria for the study.

Missing data were not imputed, except for the biologic treatment start/stop date. Treatments missing the start or stop month were excluded from analyses. When days of treatment were missing, the first day of the month was imputed. McDougall Scientific performed the calculations and statistical analyses using SAS version 9.4.

## 3 RESULTS

### 3.1 Participants

A total of 1247 charts were queried from 13 Canadian sites. Of those, 1149 (92.1%) patients were included in the FAS population and 1059 (84.9%) in the PPS population, meeting all protocol inclusion criteria. The patients’ mean (SD) age was 53.9 years (13.6) (Table 1). Approximately half of the patients were male (58.0%) and most were White (75.0%). During their PsO treatment, 71.2% of patients had been exposed to at least one IL inhibitor and 53.0% to a TNF‐α inhibitor (Tables S2 and S3). At baseline, 25.8% of patients had PsA, and 35.2% had no reported comorbidities.

**TABLE 1:** Patient demographics and baseline characteristics

	FAS (n = 1149)
Age, mean (SD)	53.9 (13.6)
Sex, n (%)	
Female	483 (42.0)
Male	666 (58.0)
Race, n (%)	
American Indian or Alaska native	11 (1.0)
Asian	64 (5.6)
Black or African American	9 (0.8)
Multiple	8 (0.7)
Unknown	195 (17.0)
White	862 (75.0)
Ethnicity, n (%)	
Hispanic or Latino	10 (0.9)
Not Hispanic or Latino	809 (70.4)
Unknown	330 (28.7)
Age at psoriasis onset (years), mean (SD)	34.0 (16.0)
Years since psoriasis onset, mean (SD)	19.9 (13.3)
Height (cm), mean (SD)	170.6 (10.1)
Weight (kg), mean (SD)	91.8 (23.1)
BMI (kg/m^2^), mean (SD)	31.2 (7.6)
Total treatment duration (years), mean (SD)	4.6 (3.1)
Comorbidities, n (%)	
PsA	297 (25.8)
Crohn’s disease	11 (1.0)
Ulcerative colitis	10 (0.9)
Uveitis	4 (0.3)
Any other	602 (52.4)
None	405 (35.2)
PsO total treatment duration (years), median (range)	3.5 (1.0–16.3)

Note.

Total treatment duration = number of years between the first and the last treatment.

Abbreviations: BMI, body mass index; FAS, full analysis set; PsA: psoriatic arthritis; PsO, psoriasis; SD, standard deviation.

### 3.2 Time to treatment change

**FIGURE 1:**
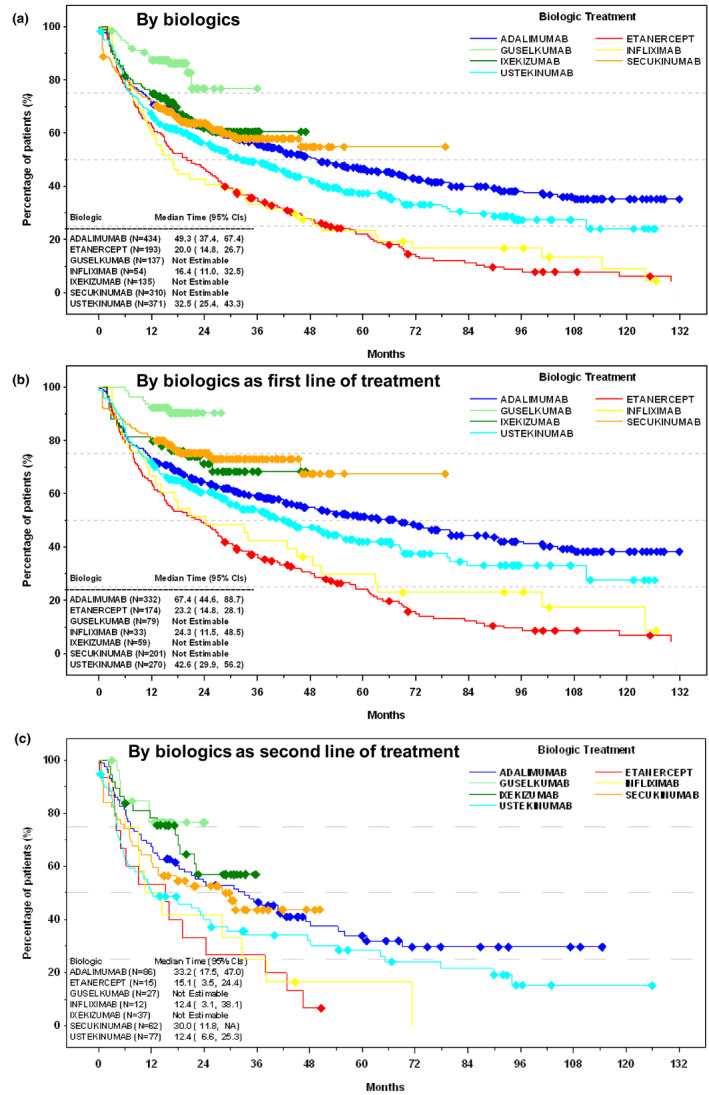
Time to treatment change, including switching, discontinuation, dose escalation, and interval change (both increasing and decreasing). (a) By biologics. (b) By biologics as first line of treatment. (c) By biologics as second line of treatment. Diamond symbols indicate censored patients; treatment for these patients was ongoing at the time of chart review, and time to treatment change was not estimable. Brodalumab is not shown due to the low number of patients using it (n = 4). CI, confidence interval; NA, not applicable.

Overall, the median time to first treatment change, including switching, discontinuation, dose escalation, and interval change (both increasing and decreasing), was 49.1 months (95% CI, 42.8–57.1 months) (Figure S1). Adalimumab showed a median time to first treatment change similar to the overall value (median time = 49.3 months; 95% CI, 37.4–67.4). Ustekinumab median time was 32.5 months (95% CI, 25.4–43.3), followed by etanercept 20.0 months (95% CI, 14.8–26.7), and infliximab 16.4 months (95% CI, 11.0–32.5) (Figure 1a). Median time to treatment change was not estimable for secukinumab, ixekizumab, and guselkumab due to the number of ongoing treatments.

By biologics, as first line of treatment, the median times to first treatment change were slightly longer than by biologics overall (Figure 1b). Adalimumab had the longest median time to first treatment change (median time, 67.4 months; 95% CI, 44.6–88.7). Ustekinumab median time was 42.6 months (95% CI, 29.9–56.2), etanercept 23.2 months (95% CI, 14.8–28.1), and infliximab 24.3 months (95% CI, 11.5–48.5). The sensitivity analysis conducted on the PPS population showed that the times to first treatment change were slightly longer than with the FAS population (adalimumab, 75.6 months [95% CI, 53.3–101.1]; and ustekinumab, 51.1 months [95% CI, 37.7–79.7]) (Figure S2).

As second line of treatment, adalimumab also had the longest median time to treatment change (median time, 33.2 months; 95% CI, 17.5–47.0), while secukinumab median time was 30.0 months (95% CI, 11.8–not applicable months) (Figure 1c). Etanercept, infliximab, and ustekinumab showed a similar median time to subsequent treatment change (12.0–15.0 months). Values for ixekizumab and guselkumab were not estimable due to the number of ongoing treatments.

### 3.3 Nature of treatment change

Half of the patients (49.3%) had a treatment change during the first line of treatment and 59.8% during the second line of treatment (Table 2). Among these, the most reported types of treatment change were interval change (44.2% for first line and 41.8% for second line) and switching to another biologic (34.1% for first line and 37.6% for second line), whereas the main reported primary reason for switches was a lack of efficacy (67.3% for the first line and 67.7% for the second line). The AE counted for approximatively 6% of patients who switched treatment.

**TABLE 2:** Nature of treatment change by first and second line of treatment (full analysis set population)

Treatment change, n (%)		Primary type of treatment change,^a^ n (%)		Primary reason for treatment change,^a^ n (%)	
First line of treatment					
No	583 (50.7)	Discontinuation of biologics	30 (5.3)	Adverse events	38 (6.7)
Yes	566 (49.3)	Dose change	45 (8.0)	Flare in comorbidity	9 (1.6)
		Interval change	250 (44.2)	Lack of efficacy	380 (67.3)
		Other	35 (6.2)	Other	53 (9.4)
		Switching to another biologic	193 (34.1)	Patient lost to follow‐up	2 (0.4)
		Unknown	13 (2.3)	Patient request	17 (3.0)
				Safety concerns	5 (0.9)
				Unknown	61 (10.8)
Second line of treatment					
No	127 (40.2)	Discontinuation of biologics	17 (9.0)	Adverse events	12 (6.3)
Yes	189 (59.8)	Dose change	13 (6.9)	Flare in comorbidity	2 (1.1)
		Interval change	79 (41.8)	Lack of efficacy	128 (67.7)
		Other	8 (4.2)	Other	16 (8.5)
		Switching to another biologic	71 (37.6)	Patient lost to follow‐up	0 (0)
		Unknown	1 (0.5)	Patient request	11 (5.8)
				Safety concerns	1 (0.5)
				Unknown	19 (10.0)

Note

Changes included switching, discontinuation, dose escalation, and interval change.

^a^ Percentages of “yes”, patients who had treatment change.

**FIGURE 2:**
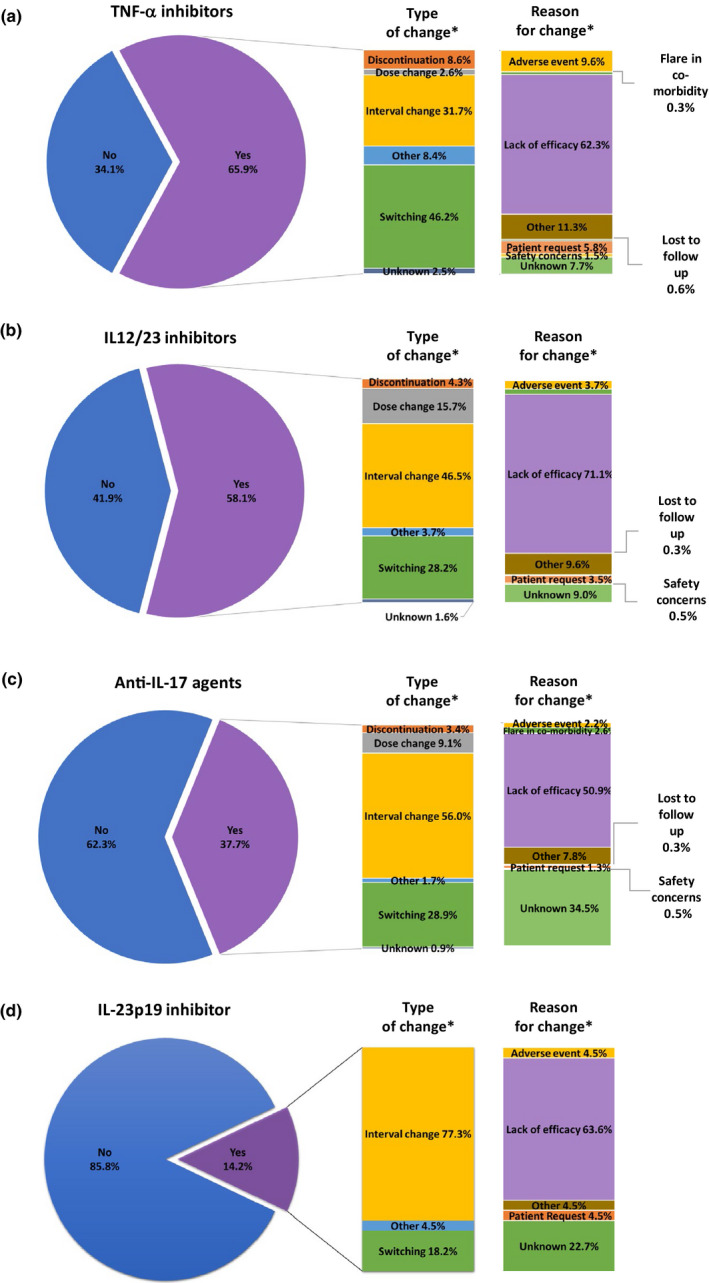
Nature of treatment change by drug class: primary type of treatment change and primary reason for treatment change. (a) Tumor necrosis factor (TNF)‐α inhibitors. (b) Interleukin (IL)‐12/23 inhibitor. (c) Anti‐IL‐17 agents. (d) IL‐23p19 inhibitor. Changes included switching, discontinuation, dose escalation, and interval change (both increasing and decreasing). Drug classes were: TNF‐α inhibitors = etanercept, adalimumab, and infliximab; IL‐12/23 inhibitor = ustekinumab; anti‐IL‐17 agents = secukinumab, ixekizumab, and brodalumab; and IL‐23p19 inhibitor = guselkumab. *Percentages of “yes”, patients who had treatment change. Reasons with percentages below 1% are not shown.

A few trends were observed based on biologics’ market entry for PsO treatment and drug selection (Table S1). Observed by drug class, patients receiving agents that had been available in Canada for a longer period of time were more likely to change treatment than those receiving newer agents (TNF‐α inhibitors, 65.9%; IL‐12/23 inhibitor, 58.1%; anti‐IL‐17 agents, 32.7%; and IL‐23p19 inhibitor, 14.2%) (Figure 2). For TNF‐α inhibitors, switching to another biologic (46.2%) was more frequent than interval change (increasing and decreasing) (31.7%), whereas the opposite was true for the IL‐12/23 inhibitor (28.2% and 46.5%, respectively) and anti‐IL‐17 agents (28.9% and 56.0%, respectively). For the IL‐23p19 inhibitor, 77.3% had an interval change. Lack of efficacy as the primary reason for switching treatment ranged from 50.9% for anti‐IL‐17 agents to 71.1% for the IL‐12/23 inhibitor. The AE were reported as the primary reason for treatment switch in 9.6% of patients treated with TNF‐α inhibitors and in less than 5% for other drug classes.

### 3.4 Number and sequence of biologic treatments for PsO

**FIGURE 3:**
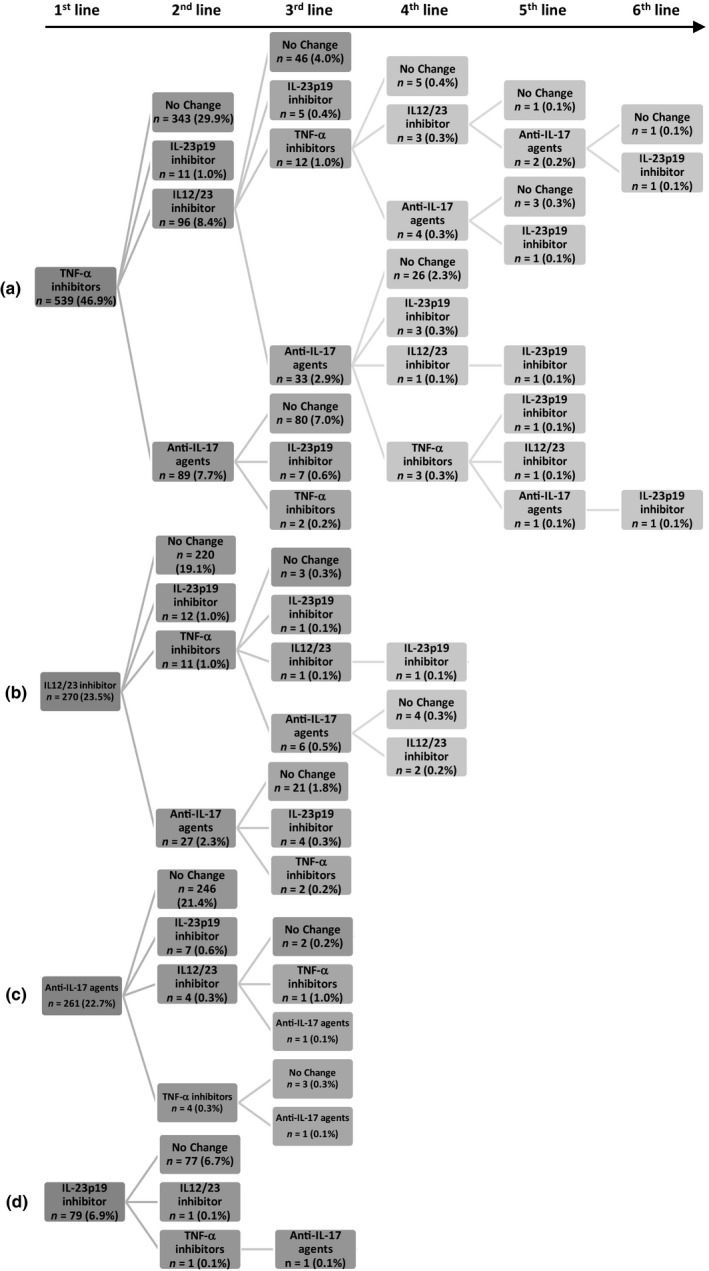
Treatment sequence by drug class. (a) Tumor necrosis factor (TNF)‐α inhibitors. (b) Interleukin (IL)‐12/23 inhibitor. (c) Anti‐IL‐17 agents. (d) IL‐23p19 inhibitor. Drug classes were: TNF‐α inhibitors = etanercept, adalimumab, and infliximab; IL‐12/23 inhibitor = ustekinumab; anti‐IL‐17 agents = secukinumab, ixekizumab, and brodalumab; and IL‐23p19 inhibitor = guselkumab.

Overall, the mean (SD) number of biologic treatments for PsO was 1.4 (0.9). The majority of patients (72.5%) received one line of treatment, and a maximum of seven lines of treatment (0.2%) was reported (Table S4). Another trend related to biologics’ market entry for PsO treatment, not surprisingly was the longer an agent’s availability on the Canadian market for the treatment of PsO, the higher the number of patients used it as first class (from 46.9% taking TNF‐α inhibitors to 6.9% taking IL‐23p19 inhibitor), and the higher the number of treatment lines (up to six drug class lines for patients who initiated treatment with TNF‐α inhibitors) (Table S1, Figure 3).

### 3.5 Drug survival

**FIGURE 4:**
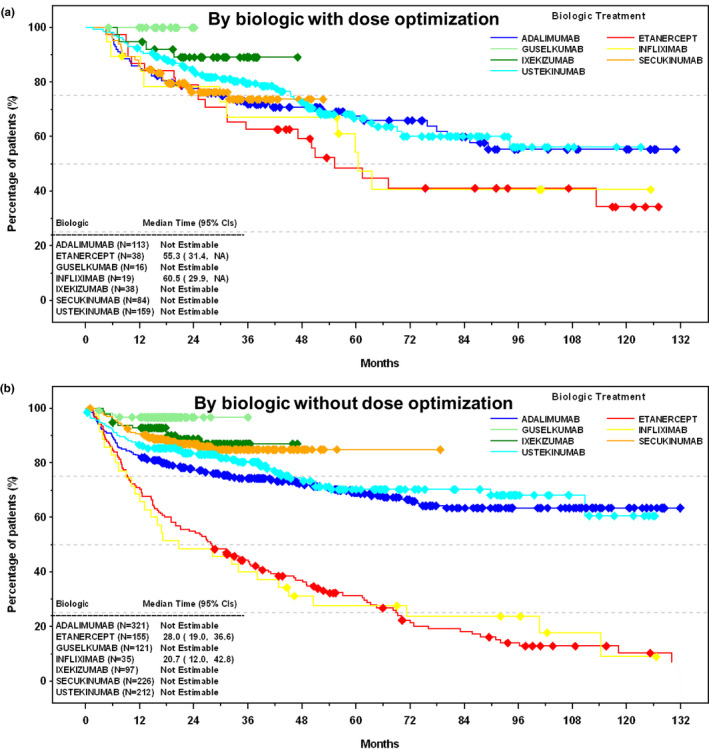
Drug survival. (a) By biologic with dose optimization. (b) By biologic without dose optimization. Drug survival defined as the number of days until discontinuation of a biologic, and dose optimization defined as dose escalation or interval change. Diamond symbols indicate censored patients; treatment for these patients was ongoing at the time of chart review. Brodalumab is not shown due to the low number of patients using it (n = 4). NA, not available.

Etanercept and infliximab showed a similar median drug survival (32–34 months) (Figure S3), which was higher for patients who had a dose optimization, namely dose escalation or interval change (55–61 months) (Figure 4a) versus for those without dose optimization (21–28 months) (Figure 4b). A large number of censored patients (ongoing treatments) prevented the estimation of drug survival median time for adalimumab, guselkumab, ixekizumab, secukinumab, and ustekinumab. However, as shown in Figure 4, the survival curves for adalimumab and ustekinumab tended to level off, especially after 48 months, and varied slightly among patients who had or did not have a dose optimization (~60% of patients remained on drug after 132 months).

## 4 DISCUSSION

This retrospective study, spanning 15 years of data (2005–2019) and four classes of biologics, provides insights on real‐world treatment patterns of commonly used biologics in Canadian patients with moderate‐to‐severe chronic plaque PsO.

Unique to this study was the assessment of time to treatment change, which included switching, discontinuation, dose escalation, and interval change. This adds to our understanding of how dose optimization influences drug survival.^19^, ^20^, ^23^, ^25^, ^29^, ^33^, ^34^, ^35^, ^36^ Our results showed that adalimumab had the longest median time to first treatment change. Etanercept had the shortest median time to first treatment change, by biologics and by biologics as first line of treatment, whereas ustekinumab had the shortest median time as second line of treatment. Of note, due to the number of ongoing treatments, the median time to first treatment change was not estimable for secukinumab, ixekizumab, and guselkumab.

Overall, half of the patients had a treatment change, and among those who had changes, more than 75% had either an interval change or a switch to another biologic. These results emphasize the unmet need in PsO to have biologics with effective on‐label dosing. On a total population basis, our results on interval change (21.8%) are similar to a real‐world observational study focusing on dose adjustments (20.0%).^19^ In a recent, shorter real‐world study from a single center observing plaque PsO patients taking guselkumab, 11.2% were reported to have a dose interval change.^9^

Exploring this unmet need in PsO to have biologics with effective on‐label dosing, in a currently recruiting, multi‐country, prospective, observational cohort study of patients with moderate‐to‐severe chronic plaque psoriasis, the VALUE study (NCT03982394),^37^ one of the primary end‐points is time to first treatment change, identified as time to either treatment discontinuation, dose escalation, or dose interval shortening. This study will provide additional real‐world data on treatment patterns worldwide, including data on the recently approved IL‐23 p19 inhibitor, risankizumab.

Our results on drug switching are aligned with those from Iskandar *et al*.^15^ and Esposito *et al*.^19^ who reported 17.5% and 14.0%, respectively, of real‐world PsO patients who switched to another biologic. As a trend related to biologics’ market entry for PsO treatment, the longer the availability of a drug class in Canada for PsO treatment, the higher likelihood to switch to another biologic as the primary type of treatment change, and vice versa for the interval change. These results are not surprising since the availability of new biologics have increased the likelihood of drug switching.^6^, ^8^ However, it is also recognized that not all switches lead to condition improvement, and additional data are required to make more specific recommendations.^7^

On a total population basis, our findings on lack of efficacy show similarities with those reported by Kishimoto *et al*.^25^ with regards to the TNF‐α inhibitors and anti‐IL‐17 agents, but are higher with regards to the IL‐12/23 inhibitor (ustekinumab). Biologic treatment switches and discontinuations due to AE have been reported in a real‐world setting to be roughly between 2% and 6%,^15^, ^19^, ^23^, ^38^, ^39^ similar to our findings. Also similar to our findings is the higher proportion of patients on TNF‐α inhibitors who switched/discontinued treatment due to AE versus other biologic classes, which has been attributed more frequently to infliximab in some publications.^25^, ^30^, ^39^ By drug class populations, our results suggest that the longer a drug class has been available for treating PsO, more often a treatment switch may be attributable to AE.

A relation between drug class and the number of treatment lines, as well as the proportions of patients who changed lines was notable. From a first line to a second line of treatment, 36.4% switched from TNF‐α inhibitors, 18.5% from IL‐12/23 inhibitor, 5.7% from anti‐IL‐17 agents, and 2.5% from IL‐23p19 inhibitor. Notwithstanding the initial treatment or the drug class lines, almost all patients who switched to the IL‐23p19 inhibitor at some point did not further switch to another class during the study, which may be partly due to the recent availability of this class in Canada.

Estimable for etanercept and infliximab, drug survival was approximately twice as long when stratified by patients who went through dose optimization (i.e., dose escalation or interval change) than patients who did not, supporting the validity of dose escalation or interval change as an effective treatment optimization strategy.^8^ The survival curve of adalimumab after 48 months was similar to the curve for ustekinumab, which is often found to have a higher survival curve among biologics, especially in studies with shorter observation times, including two Canadian studies.^20^, ^21^, ^23^, ^25^, ^30^, ^33^, ^34^, ^35^, ^36^ Our survival curves show that after 132 months of treatment, approximately 60% of patients using adalimumab and ustekinumab remained on treatment. The longer time to first treatment change observed with adalimumab versus other biologics studied and the relatively high survival curve of adalimumab compared with other publications^19^, ^20^, ^21^, ^23^, ^25^, ^30^, ^33^, ^34^, ^35^, ^36^ might be partly explained by the use of combination therapy that may be more prevalent in Canada for adalimumab compared with other agents, for medication coverage reasons, or because adalimumab was used for treating other conditions, such as PsA, inflammatory bowel diseases, or hidradenitis suppurativa.^40^

This chart review was limited due to the review period focusing mostly on the last few years. No effectiveness data were collected. The introduction of new biologics may have impacted drug survival patterns as patients may have switched earlier if/when new options became available. Also, treatment goals and patterns evolved over time. The longer a biologic has been on the market the higher the likelihood that is has been used as a first treatment. Limited long‐term data were available for newer biologics; therefore, the estimation of treatment changes could not be performed. Restricting inclusion of patients with treatment initiated per label dose may not fully reflect clinical practice. Some sites could not provide the requested number of charts to meet the inclusion criteria within the study time frame; therefore, these sites went sequentially backwards in 2018 until they reached 100 charts. Despite this approach, some sites were unable to provide 100 charts, mainly due to current therapy lasting <1 year. Due to the retrospective nature of this study, missing or incomplete information was also a limitation. Future investigations should explore temporal treatment changes at a deeper level.

Novel from this study was the assessment of time to first treatment change – including switching, discontinuation, dose escalation, and interval change (both increasing and decreasing) – which was longer for adalimumab than ustekinumab or etanercept. The time to first treatment change could be half as long for the second line of treatment than for the first line of treatment. Specifically, in the Canadian context where dosing is usually neither decreased nor intervals increased as much as in Europe,^11^, ^41^, ^42^ this study provides long‐term data on four classes of biologics, helping to close a knowledge gap on treatment pattern practices in a real‐world setting in patients with moderate‐to‐severe chronic plaque PsO.

## CONFLICTS OF INTEREST

Dr Gooderham received honoraria from AbbVie, Amgen, Akros, Arcutis, BMS, Boehringher Ingelheim, Celgene, Dermira, Dermavant, Eli Lilly, Galderma, GSK, Janssen, Kyowa Kirin, Merck, Medimmune, Novartis, Pfizer, Roche, Regeneron, UCB, and Valeant/Bausch; Dr Lynde received honoraria from AbbVie, Amgen, Arcutis, Bausch Health, BMS, Boehringer Ingelheim, Celgene, Demira, Eli Lilly, Galderma, GSK, Janssen, Kyowa, Merck, Novartis, Pfizer, Regeneron, Roche, Sun Pharma, UCB, and Valeant; Dr Turchin received honoraria from AbbVie, Amgen, Arcutis, Bausch Health, Boehringer Ingelheim, Celgene, Eli Lilly, Galderma, Janssen, Novartis, Pfizer, and UCB; M. Avadisian and M. Labelle are employees of AbbVie and may have received stocks and/or stock options; Dr Papp received honoraria from AbbVie, Akros, Amgen, Arcutis, Bausch Health/Valeant, Boehringer Ingelheim, Bristol Myer Squibb, Celgene, Cipher, Coherus, Dermira, Eli Lilly, EMD Serono, Galderma, GSK, Janssen, Kyowa Hakko Kirin, Leo Pharma, Merck (MSD), Merck Serono, Merck KG, Mitsubishi Pharma, Novartis, Pfizer, PRCL Research, Regeneron, Roche, Sanofi‐Aventis/Genzyme, Sun Pharma, Takeda, and UCB.

## Supporting information

Supplementary Material
