# Supplementary material for: Real‐world, long‐term treatment patterns of commonly used biologics in Canadian patients with moderate‐to‐severe chronic plaque psoriasis
Source: J Dermatol. 2021 Nov 7;49(1):95–105. doi: 10.1111/1346-8138.16214 (PMC9298415; doi:10.1111/1346-8138.16214)

Real-world, long-term treatment patterns of commonly used biologics in Canadian patients with moderate-to-severe chronic plaque psoriasis

Melinda J. GOODERHAM, Charles LYNDE, Irina TURCHIN, Miriam AVADISIAN, Melanie LABELLE, Kim A. PAPP

Supplemental Tables

Supplemental Table 1. Summary of PsO Biologics Canadian On-Label Dosing

PsO Biologic	Canadian On-Label Dosing	Product Entry on the Canadian Market for PsO Treatment
Etanercept	50 mg twice weekly for 3 months, then 50 mg ew	2004
Infliximab	5 mg/kg wk 0, 2, 6 then Q8W	2006
Adalimumab	80 mg wk 0, 40 mg Q2W starting at wk 1	2008
Ustekinumab	45 mg for <100 kg; 90 mg for >100 kg; wk 0, 4 then Q12W; for patients who inadequately respond to Q12W, consideration may be given to treating Q8W	2009
Secukinumab	300 mg wk 0, 1, 2, 3, and 4 then monthly dosing	2015
Ixekizumab	160 mg wk 0, then 80 mg wk 2, 4, 6, 8,10,12 then 80 mg Q4W	2016
Brodalumab	210 mg wk 0, 1, 2 then Q2W	2018
Guselkumab	100 mg wk 0, 4 then Q8W	2018

EW, every week; PsO, psoriasis; Q2W, every 2 weeks; Q4W, every 4 weeks; Q8W, every 8 weeks; Q12W, every 12 weeks; wk, week.

Supplemental Table 2. Non-biologic treatments for psoriasis taken by more than 1% of patients in decreasing order - By WHO-DD

	Non-Biologics	
**ATC Chemical Subgroup**	**Patients (*n* = 1149)**	**Events (*n* = 5315)**
All non-biologic treatments	1049 (91.3%)	5315 (100.0%)
Other antipsoriatics for topical use	584 (50.8%)	923 (17.4%)
Other immunosuppressants	506 (44.0%)	764 (14.4%)
Corticosteroids, potent (group III)	421 (36.6%)	802 (15.1%)
Corticosteroids, very potent (group IV)	365 (31.8%)	525 (9.9%)
All other therapeutic products - Not available	315 (27.4%)	449 (8.4%)
Retinoids for treatment of psoriasis	263 (22.9%)	317 (6.0%)
Corticosteroids, weak (group I)	159 (13.8%)	184 (3.5%)
Selective immunosuppressants	145 (12.6%)	159 (3.0%)
Calcineurin inhibitors	130 (11.3%)	202 (3.8%)
Agents for dermatitis, excluding corticosteroids	119 (10.4%)	148 (2.8%)
Corticosteroids, potent, other combinations	108 (9.4%)	122 (2.3%)
Corticosteroids, moderately potent (group II)	73 (6.4%)	83 (1.6%)
Tars	68 (5.9%)	93 (1.7%)
Folic acid and derivatives	44 (3.8%)	49 (0.9%)
Glucocorticoids	44 (3.8%)	100 (1.9%)
Imidazole and triazole derivatives	39 (3.4%)	46 (0.9%)
Corticosteroids, dermatological preparations – Not available	30 (2.6%)	30 (0.6%)
Other antifungals for topical use	29 (2.5%)	29 (0.5%)
Salicylic acid preparations	23 (2.0%)	25 (0.5%)
Uncoded – Not available	21 (1.8%)	21 (0.4%)
Agents for dermatitis, excluding corticosteroids – Not available	20 (1.7%)	24 (0.5%)
Soft paraffin and fat products	16 (1.4%)	17 (0.3%)
Other antibiotics for topical use	14 (1.2%)	15 (0.3%)
Corticosteroids, very potent, other combinations	14 (1.2%)	18 (0.3%)
Medicated shampoos	14 (1.2%)	15 (0.3%)
Piperazine derivatives	13 (1.1%)	14 (0.3%)
Corticosteroids, plain	13 (1.1%)	13 (0.2%)

ATC Chemical Subgroup "Not Available" meant that medication's chemical subgroup was not available in WHO-DD. Concomitant Medications were coded using WHO-DD C3 SEP 2019.
ATC, Anatomical Therapeutic Chemical; WHO-DD, World Health Organizations Drug Dictionary

Supplemental Table 3. Biologic treatments for psoriasis - By WHO-DD

**ATC therapeutic class**<br>**ATC chemical subgroup**	**Patients**<br>**(*n* = 1149)**	**Events**<br>**(*n* = 2659)**
**All biologic treatments**	1149 (100.0%)	2659 (100.0%)
**Antineoplastic agents**	6 (0.5%)	6 (0.2%)
Monoclonal antibodies	5 (0.4%)	5 (0.2%)
Protein kinase inhibitors	1 (0.1%)	1 (0.0%)
**Antipsoriatics**	4 (0.3%)	4 (0.2%)
Not available	4 (0.3%)	4 (0.2%)
**Immunosuppressants**	1149 (100.0%)	2643 (99.4%)
Interleukin inhibitors	818 (71.2%)	1483 (55.8%)
Selective immunosuppressants	42 (3.7%)	52 (2.0%)
TNF-α inhibitors	609 (53.0%)	1108 (41.7%)
**Investigational drug**	5 (0.4%)	6 (0.2%)
Not available	5 (0.4%)	6 (0.2%)

Biologic treatments were coded using WHO-DD C3 September 2019.
ATC, anatomical therapeutic chemical; TNF, tumor necrosis factor; WHO-DD, World Health Organisation Drug Dictionary.

Supplemental Table 4. Line of biologic treatments for psoriasis

	**FAS (*n* = 1149)**
**Number of biologic treatments**	
Mean (SD)	1.4 (0.86)
Median	1.0
Min, max	1, 7
**Lines of treatment**	
1	833 (72.5%)
2	190 (16.5%)
3	85 (7.4%)
4	29 (2.5%)
5	5 (0.4%)
6	5 (0.4%)
7	2 (0.2%)

FAS, full analysis set; SD, standard deviation.

Supplemental Figure Legends

Supplemental Figure 1. Time to first treatment change overall; change including switching, discontinuation, dose escalation, and interval change (both increasing and decreasing). CI, confidence interval.

Supplemental Figure 2. Time to first treatment change; change including switching, discontinuation, dose escalation, and interval change (both increasing and decreasing) – Sensitivity analysis. (a) Overall. (b) By biologics as first line of treatment.

Supplemental Figure 3. Drug survival by biologic. Drug survival defined as the number of days until discontinuation of a biologic. Diamond symbols indicate censored patients; treatment for these patient was ongoing at the time of chart review. Brodalumab is not shown due to the low number of patients using it (n = 4). NA, not available.

Supplemental Figure 1

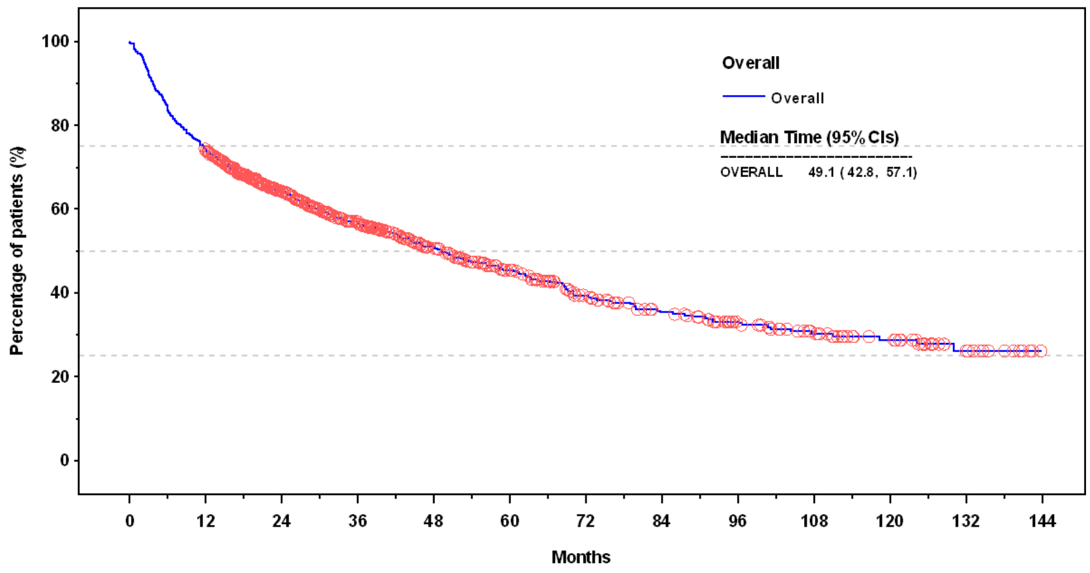

Supplemental Figure 2

(a)

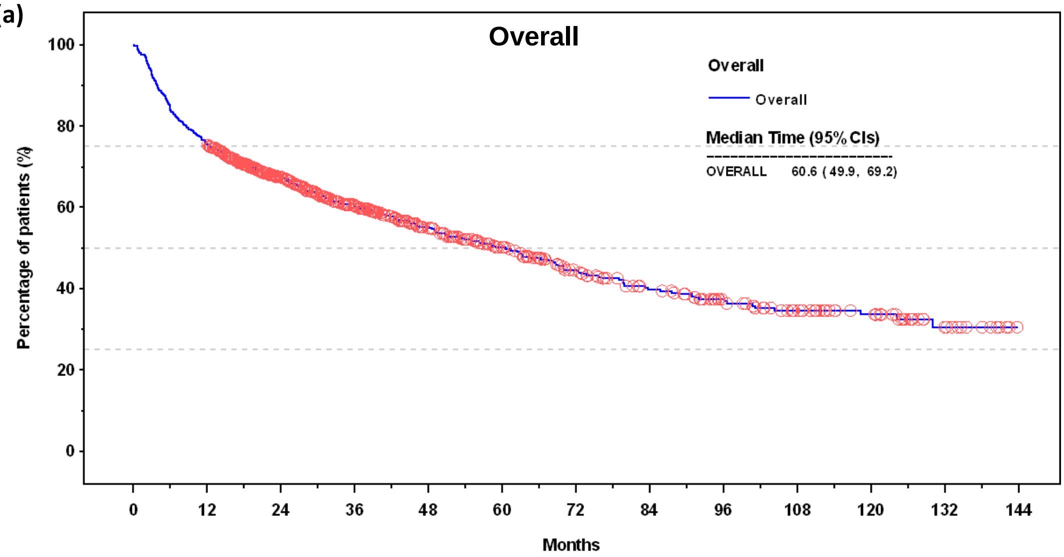

(b)

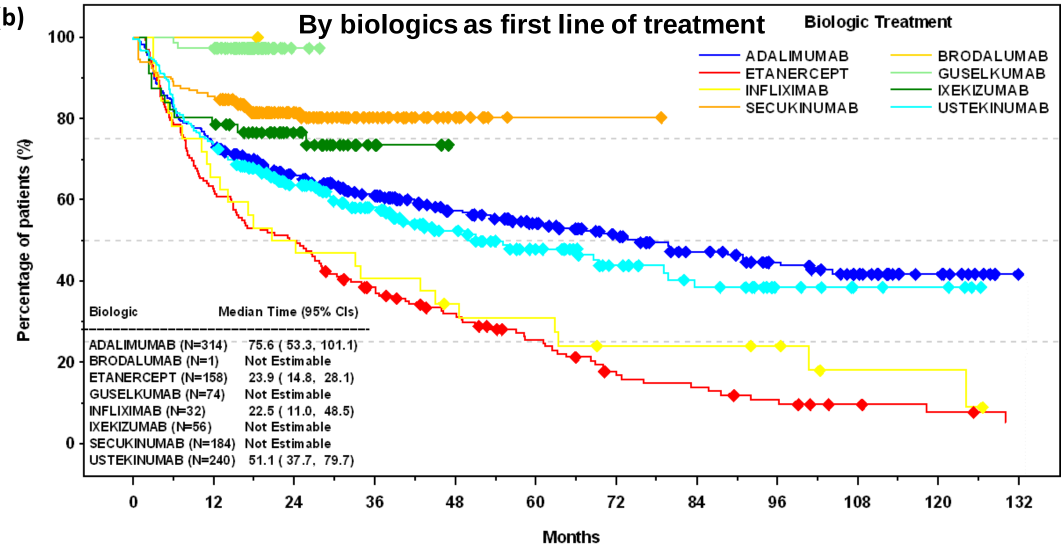

Supplemental Figure 3

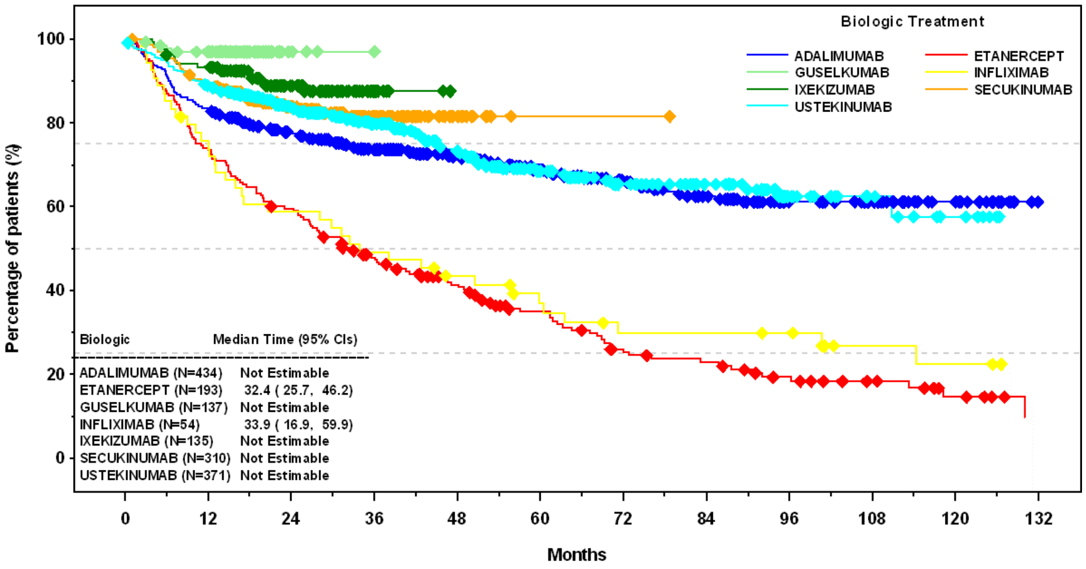

Supplement: Supplementary file 1 — Supplementary Material [file JDE-49-95-s001.pdf]
